# Landiolol in patients with septic shock resident in an intensive care unit (LANDI-SEP): study protocol for a randomized controlled trial

**DOI:** 10.1186/s13063-018-3024-6

**Published:** 2018-11-19

**Authors:** Martin Unger, Andrea Morelli, Mervyn Singer, Peter Radermacher, Sebastian Rehberg, Helmut Trimmel, Michael Joannidis, Gottfried Heinz, Vladimír Cerny, Pavel Dostál, Christian Siebers, Fabio Guarracino, Francesca Pratesi, Gianni Biancofiore, Massimo Girardis, Pavla Kadlecova, Olivier Bouvet, Michael Zörer, Barbara Grohmann-Izay, Kurt Krejcy, Christoph Klade, Günther Krumpl

**Affiliations:** 1 AOP Orphan Pharmaceuticals AG, Wilhelminenstraße 91/II f, 1160 Vienna, Austria; 2 Department of Anesthesiology and Intensive Care, University Hospital La Sapienza, Policlinico Umberto I, Rome, Italy; 3 Intensive Care Medicine, University College London, London, UK; 4 Institute of Anesthesiologic Pathophysiology and Process Engineering, Ulm University Hospital, Ulm, Germany; 5 Department of Anesthesiology, Intensive Care, Emergency and Pain Medicine, University Hospital Greifswald, Greifswald, Germany; 6 Department of Anesthesiology, Emergency Medicine and General Intensive Care, State Hospital Wiener Neustadt, Wiener Neustadt, Austria; 7 Division of Emergency Medicine and Intensive Care, Department Internal Medicine, Medical University Innsbruck, Innsbruck, Austria; 8 Department of Internal Medicine II, Division of Cardiology, Intensive Care Unit, Medical University General Hospital, Vienna, Austria; 9 Department of Anesthesiology, Perioperative Medicine and Intensive Care, Masaryk Hospital, Usti Nad Labem, Czech Republic; 10 Department of Anesthesiology, Resuscitation and Intensive Medicine, University Hospital Hradec Králové, Hradec Králové, Czech Republic; 11 Department of Anesthesiology, University Hospital Munich, Munich, Germany; 12 Department of Anesthesiology and Resuscitation 5, Azienda Ospedaliero Universitaria Pisana, Pisa, Italy; 13 Department of Anesthesiology and Resuscitation 6, Azienda Ospedaliero Universitaria Pisana, Pisa, Italy; 14 Division of Transplant Anesthesia and Critical Care, University School of Medicine Pisa, Pisa, Italy; 15 Department of Anesthesia and Intensive Care, University Hospital of Modena, Modena, Italy; 16 Aixial s.r.o, Brno, Czech Republic; 17 Amomed Pharma GmbH, Vienna, Austria

**Keywords:** Septic shock, Sepsis, Beta-blocker, Landiolol, Tachycardia, Randomized controlled trial

## Abstract

**Background:**

In patients with septic shock, the presence of an elevated heart rate (HR) after fluid resuscitation marks a subgroup of patients with a particularly poor prognosis. Several studies have shown that HR control in this population is safe and can potentially improve outcomes. However, all were conducted in a single-center setting. The aim of this multicenter study is to demonstrate that administration of the highly beta1-selective and ultrashort-acting beta blocker landiolol in patients with septic shock and persistent tachycardia (HR ≥ 95 beats per minute [bpm]) is effective in reducing and maintaining HR without increasing vasopressor requirements.

**Methods:**

A phase IV, multicenter, prospective, randomized, open-label, controlled study is being conducted. The study will enroll a total of 200 patients with septic shock as defined by The Third International Consensus Definitions for Sepsis and Septic Shock criteria and tachycardia (HR ≥ 95 bpm) despite a hemodynamic optimization period of 24–36 h. Patients are randomized (1:1) to receive either standard treatment (according to the Surviving Sepsis Campaign Guidelines 2016) and continuous landiolol infusion to reach a target HR of 80–94 bpm or standard treatment alone. The primary endpoint is HR response (HR 80–94 bpm), the maintenance thereof, and the absence of increased vasopressor requirements during the first 24 h after initiating treatment.

**Discussion:**

Despite recent studies, the role of beta blockers in the treatment of patients with septic shock remains unclear. This study will investigate whether HR control using landiolol is safe, feasible, and effective, and further enhance the understanding of beta blockade in patients with septic shock.

**Trial registration:**

EU Clinical Trials Register; EudraCT, 2017-002138-22. Registered on 8 August 2017.

**Electronic supplementary material:**

The online version of this article (10.1186/s13063-018-3024-6) contains supplementary material, which is available to authorized users.

## Background

In the early phase of septic shock, overwhelming inflammation leads to vasodilation and capillary leakage, which decreases cardiac output due to both absolute and relative hypovolemia [1–3]. These alterations trigger massive sympathetic activation in the attempt to maintain vital organ perfusion. Tachycardia and vasoconstriction are the hallmarks of this activation and compensate for systemic vasodilatation [4]. In the very early phase of the septic insult, tachycardia is the main compensatory mechanism to maintain cardiac output despite the reduction of preload. Accordingly, current sepsis guidelines recommend intravascular fluid administration as the first step to counteract hypotension [4]. Compensatory tachycardia implies preserved baroreceptor and chemoreceptor activity, thus the majority of patients with sepsis rapidly respond to volume administration with a reduction of tachycardia.

However, some patients with sepsis continue to have an elevated heart rate (HR) despite adequate fluid resuscitation. This elevated HR reflects sympathetic overstimulation resulting from dysregulation of the autonomic nervous system [5–12] in addition to the effect of exogenous catecholamines [7].

Elevated HR has been associated with a poor outcome, but it is unclear whether it is a surrogate of disease severity or whether it plays a pathophysiological role that could be treated to improve patient outcomes [13–16].

Beta-blockers are potential candidates to control HR and numerous animal models provide a rationale for their use during sepsis [17–24]. Despite concerns of hemodynamic decompensation, recent clinical studies using esmolol in patients with sepsis [10, 25–34] suggest that control of HR can be safely achieved with beta1-selective beta-blockers. These studies reported a decrease in HR with limited reduction of cardiac output, improved stroke volume and lactate levels, and stabilization or improvement of organ dysfunction [10, 28–30, 32]. Furthermore, the combined use of beta-blockers and vasopressors appears to be safe and does not appear to increase the need for vasopressor support or impair microcirculation [26, 27, 34]. However, all of the previously reported studies were conducted in single centers with relatively small sample sizes and only one study included a Caucasian population.

Landiolol, the beta-blocker used in our study, is a highly beta1-selective, ultrashort-acting beta-blocker that could be ideally suited for the treatment of critically ill patients due to its limited hypotensive effect [35–38].

The aim of this multicenter, prospective, controlled study is to demonstrate that the administration of the ultrashort-acting beta-blocker landiolol in patients with septic shock and persistent tachycardia (HR ≥ 95 beats per minutes [bpm]) is effective in reducing and maintaining HR without increasing vasopressor requirements.

## Methods/Design

### Study design and objective

This is a phase IV, multicenter, prospective, randomized, open-label, controlled study on landiolol in a septic shock population (as defined by The Third International Consensus Definitions for Sepsis and Septic Shock criteria [39]) hospitalized in an intensive care unit (ICU). The study duration is expected to be 24 months from first patient enrolled until completion of the final visit for the last patient. Participating centers are listed in Table 1.

**Table 1:** List of participating centers and ethics committee approvals

Participating center	PI	Central Ethics committee	Reference number	Approval date
Medical University Vienna, Department of Internal Medicine II. Division of Cardiology	Gottfried Heinz, MD	Ethics Committee, Medical University Vienna	ECS 1805/2017	15 September 2017
Medical University Innsbruck, Division of Emergency Medicine and Intensive Care, Department Internal Medicine	Michael Joannidis, MD			
State Hospital Wiener Neustadt, Department of Anesthesiology, Emergency Medicine and General Intensive Care	Helmut Trimmel, MD, MSc			
University Hospital Greifswald, Department of Anesthesiology, Intensive Care, Emergency and Pain Medicine	Sebastian Rehberg, MD	Ethics Committee, University Hospital Greifswald	FFV 06/17	15 February 2018
University Hospital Munich, Department of Anesthesiology	Christian Siebers, MD			
University Hospital Hradec Králové, Department of Anesthesiology, Resuscitation and Intensive Medicine	Pavel Dostál, MD, PhD, MBA	Ethics Committee, University Hospital Hradec Králové	201801 I126M	07 November 2017
Masaryk Hospital, Department of Anesthesiology, Perioperative Medicine and Intensive Care	Vladimír Černý, MD, PhD, FCCM			
University Hospital La Sapienza, Department of Anesthesiology and Intensive Care	Andrea Morelli, MD.	Ethics Committee, University Hospital La Sapienza	4846	08 February 2018
Azienda Ospedaliero Universitaria Pisana, Department of Anesthesiology and Resuscitation 5	Fabio Guarracino, MD			
Azienda Ospedaliero Universitaria Pisana, Department of Anesthesiology and Resuscitation 6	Francesca Pratesi, MD			
University School of Medicine Pisa, Department of Anesthesiology and Transplant Intensive Care Unit	Gianni Biancofiore, MD			
University Hospital Modena, Department of Anesthesia and Intensive Care	Massimo Girardis, MD		Approval pending	

The study objective is to compare the percentage of patients with a HR response (defined as HR within the target range of 80–94 bpm) and maintenance thereof without an increase in vasopressor requirements within the first 24 h of treatment, and to further assess efficacy and safety in the two treatment arms: standard of care treatment and landiolol (landiolol group) or standard of care treatment alone (control group).

Additional file 1 contains the completed Standard Protocol Items: Recommendations for Interventional Trials (SPIRIT) checklist.

### Study population

This study will enroll a total of 200 patients with septic shock and elevated HR (≥ 95 bpm) despite a hemodynamic optimization phase of at least 24 h but a maximum of 36 h in which they received adequate fluid resuscitation and continuous vasopressor treatment (according to the Surviving Sepsis Campaign Guidelines 2016 [40]). Detailed inclusion and exclusion criteria are displayed in Table 2.

**Table 2:** Inclusion/exclusion criteria

Inclusion criteria 1. Informed consent 2. Age ≥ 18 years 3. Confirmed septic shock: a. Confirmed or suspected infection b. Acute increase of ≥ 2 points on SOFA Score c. Need for continuous vasopressor therapy to maintain a mean arterial pressure (MAP) of > 65 mmHg despite adequate fluid resuscitation d. Blood lactate > 2 mmol/L (18 mg/dL)^a^ 4. Tachycardia and/or tachyarrhythmia with heart rate ≥ 95 bpm 5. Norepinephrine infusion rate ≥ 0.2 μg/kg/min at the time of study inclusion 6. Patients must have undergone a hemodynamic optimization period of at least 24 h but a maximum of 36 h, during which period they received continuous vasopressor treatment and standard treatment for septic shock according to the SSCG 2016 guidelines ^a^Presence of blood lactate > 2 mmol/L (18 mg/dL) and increase of ≥ 2 points on SOFA score are mandatory for the diagnosis of septic shock, but must not necessarily be present at the time of study inclusion Exclusion criteria: 1. Any form of compensatory tachycardia 2. β-blocker treatment within 72 h before randomization 3. Sick sinus syndrome, or second or third degree AV block 4. Patients with any form of cardiac pacing 5. A known serious cardiovascular condition such as ischemic stroke or transient ischemic attack within the last six months, or pre-existing heart failure NYHA class III or IV 6. Cardiogenic shock 7. MAP < 65 mmHg 8. Known pulmonary hypertension 9. Known terminal illness other than septic shock with expected patient’s survival < 28 days 10. Known presence of an advanced condition to withhold life-sustaining treatment 11. Patients for whom a “Do Not Resuscitate” (DNR) order exists 12. Known sensitivity to any component of the study medication (e.g. landiolol, mannitol) 13. Participation in a clinical drug trial within 30 days before randomization 14. Any condition that, in the investigator’s opinion, makes the individual unsuitable for study participation (to be documented) 15. Pregnant or breast-feeding patients 16. Untreated pheochromocytoma	

*NYHA* New York Heart Association

### Randomization, blinding, and treatment allocation

Patients fulfilling the selection criteria are randomized in a 1:1 ratio to one of the two groups (landiolol or control) after informed consent, as required by local law, has been obtained. The presence of atrial fibrillation in the hemodynamic optimization period is used as a stratification factor for randomization. As this is an open label study, investigators and other study personnel will not be blinded to the treatment.

### Study drug

Lyophilized landiolol hydrochloride 300 mg (Rapibloc Lyo, 300 mg) is to be reconstituted in 50 mL of 0.9% NaCl to a concentration of 6 mg/mL before use.

### Treatments

#### Landiolol group

Patients in the landiolol group begin continuous infusion with landiolol within 2 h after randomization at a starting dose of 1 mcg/kg/min. The dose is to be progressively increased at increments of 1 mcg/kg/min to a maximum of 40 mcg/kg/min at intervals of at least 20 min to obtain and maintain a HR of 80–94 bpm. Landiolol must be infused continuously to maintain the target HR until one the following events occurs: discontinuation of vasopressor infusion; death; a serious adverse event (AE) attributable to the study drug that necessitates study drug discontinuation as determined by the investigator; patient discharge from the ICU; or day 28 of study participation.

#### Control group

Patients in the control group receive standard of care treatment according to the Surviving Sepsis Campaign Guidelines 2016 [40], which does not specify a target for HR control. Patients in the control group are to be withdrawn from the study if they receive beta-blocker treatment.

### Patient assessments

**Fig. 1:**
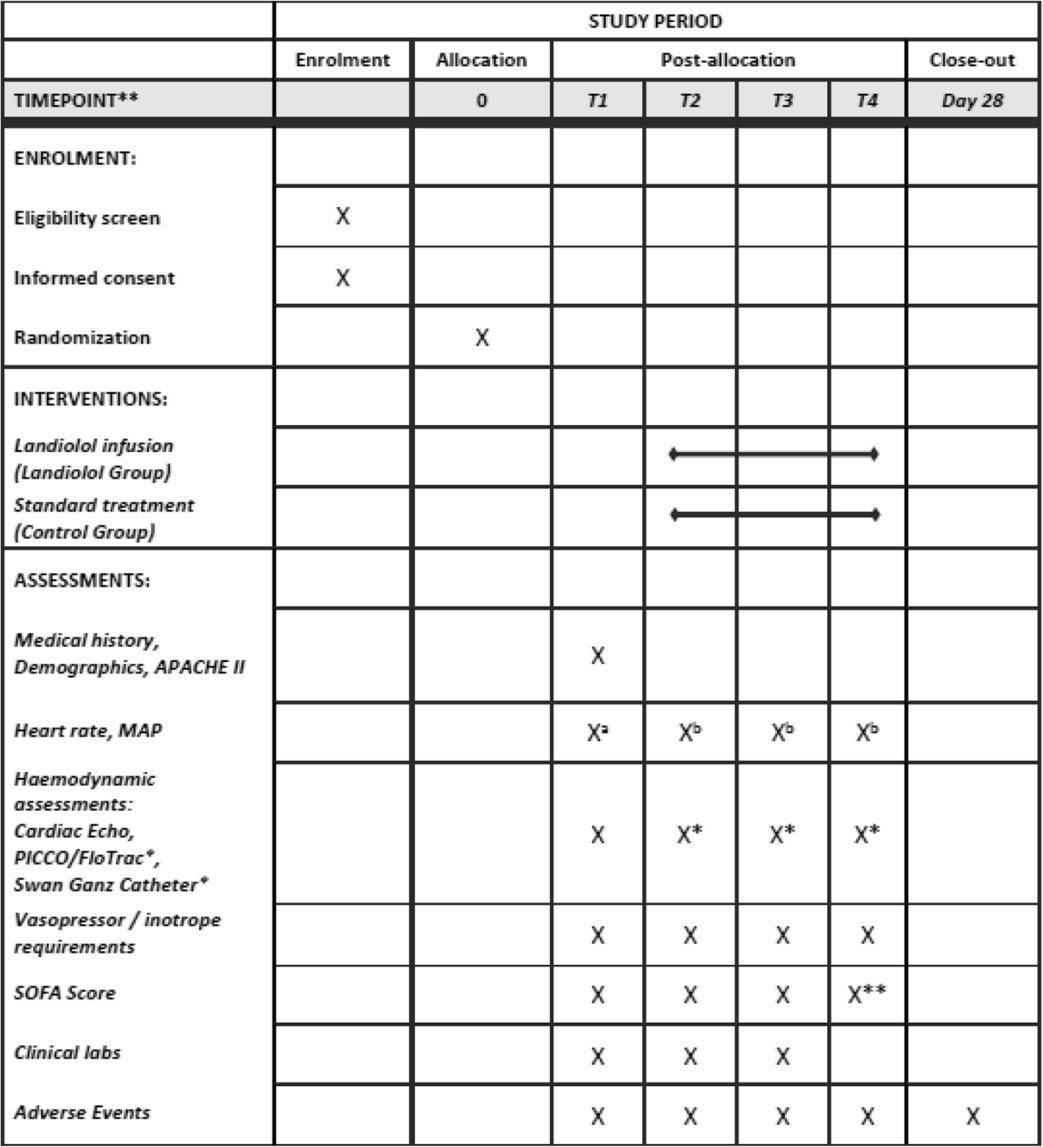
Schedule of enrolment and assessments (SPIRIT 2013 Figure)

Heart rate, blood pressure, and body temperature will be documented hourly for the first 24 h after treatment start and every 12 h thereafter in both treatment groups, and additionally at every dose change of landiolol in the landiolol group. Clinical laboratory analysis including blood gas analysis will be performed daily for the first four days of the study. Sequential Organ Failure Assessment (SOFA) score will be assessed daily for the first four days and every third day thereafter. If performed, hemodynamic parameters (CO, CI, GEDI, ELWI, PAOP, MPAP, LVEF, TAPSE, VTI) obtained by PICCO/FloTrac, Swan-Ganz catheter or Cardiac Echo will be documented. Concomitant medication and AE will be documented over the entire study period. Measurements and assessments performed in both groups are listed in Fig. 1 in the completed SPIRIT figure.

### Endpoints

The primary efficacy endpoint is HR response (HR = 80–94 bpm) and maintenance thereof without increase in vasopressor requirements during the first 24 h after treatment start.

Secondary efficacy endpoints will consist of ICU and 28-day mortality, ICU and hospital stay duration, SOFA score, and inotrope and vasopressor support requirements. Efficacy and safety endpoints are listed in Table 3.

**Table 3:** Efficacy and safety endpoints

Primary endpoint: • HR response (i.e. HR = 80–94 bpm) and maintenance thereof and no increase in vasopressor requirements during the first 24 h after treatment start Secondary endpoints: • Change in vasopressor requirements over the study period (dose and duration) • HR response (i.e. HR = 80–94 bpm) during the first 24 h after treatment start • 28-day mortality (all cause) • ICU mortality (all cause) • Duration of ICU stay (survivors/non-survivors) • Duration of hospital stay (survivors/non-survivors) • SOFA score (as long as the patient is treated with vasopressors) on days 1, 2, 3, 4, 7, 10, 13, 16, 19, 22, 25, and 28 • Daily inotropic requirements (as long as the patient is treated with vasopressors) Safety endpoints: • Incidence rate of bradycardic episodes requiring intervention • Incidence of adverse events (AE) • Incidence of serious adverse events (SAE)	

### Data collection

Data will be collected and entered into the electronic data capture system by trained study personnel (investigators and study nurses).

### Sample size

Sample size estimation is based upon the assumption that 60% of patients in the landiolol group reach the primary endpoint versus 40% of patients in the control group. The sample size of 200 patients will provide 80% power to demonstrate a statistically significant difference (upon standard level alpha = 0.05) between the treatment groups using a Chi-square test. The sample size in the study by Morelli et al. [10] was adequate considering that to detect a 20% change in HR with a power of 80% at a level of significance of alpha = 0.05, 64 patients per group would have been required. In order to detect the binary primary endpoint of our study an additional 36 patients per group are required.

### Statistical analysis

The hypothesis that Group L is superior to Group C in proportion of patients who reached the primary endpoint will be demonstrated if the lower limit of the two-sided 95% Newcombe confidence interval of difference pL-pC is above zero, where pL and pC are percentages of patients who reached the primary endpoint in Group L and Group C, respectively. *P* values based on Cochran–Mantel–Haenszel (according to SAS® terminology) will be presented together with the confidence intervals to evaluate statistical significance of association between treatment group and outcome after adjustment for the stratification group. For the purpose of exploratory analysis, the individual criteria of the primary endpoint, heart rate response (i.e. HR = 80–94 bpm) reached (also defined as secondary endpoint), heart rate response reached and maintained and no increase in vasopressor requirements during the first 24 h, will be compared separately between treatment groups. Additional subgroup and sensitivity analyses will have exploratory character and will be defined in all details in the SAP.

Secondary endpoints with continuous, ordinal, and binary variables measured at multiple time-points will be analyzed as longitudinal data by linear, ordinal logistic, logistic, or log-binomial regression models with repeated measures. Covariates used in the models will be (but not limited to) treatment, visit, stratification group, and interaction treatment/visit. For continuous variables baseline value of the outcome variable will be a covariate as well. Distribution of data and a feasibility check of planned analyses will be performed before finalization of SAP and alternative statistical methods will be defined if assumptions on the application of the planned methods are not met (e.g. ln-transformation of data, non-parametric method, or other alternative way). ICU mortality and 28-day mortality will be analyzed using the same methods as for the primary endpoint. Duration of ICU stay and duration of hospital stay (in survivors/non-survivors) will be analyzed as time-to-event data (using Kaplan–Meier curve and using log-rank test or Wilcoxon test and/or proportional hazards regression model if appropriate).

For secondary analyses, no multiplicity adjustment is planned; therefore, a higher rate of type-I error must be considered in interpretation of results of secondary analyses. The analysis of secondary endpoints/analyses can provide supportive evidence related to the primary objective, but no confirmatory conclusion based on secondary analyses can be done.

## Discussion

Despite intensive research, morbidity and mortality of patients with sepsis remain high [39]. Hence, novel therapeutic concepts are urgently needed. Recently, the use of beta-blockers during sepsis has been suggested [10, 28–30, 32, 41]. This represents a true innovation, as current guidelines recommend beta-mimetics [40]. The potential benefits of beta-blockers are most likely due to their pleotropic effects and include myocardial protection, modulation of inflammatory processes, and improvements in organ functions [3, 22, 42–44]. However, it is still unclear which patients would benefit most from this intervention.

Morelli et al. [10] (and later others [28–30, 32]) showed that a HR reduction in patients with septic shock, after adequate resuscitation with fluids, vasopressors, and inotropes, was not associated with an increase in AEs and did not trigger an increase in vasopressor support. As in the original protocol by Morelli et al. [10], the target HR in the present study is < 95 bpm, which is based on studies showing poorer patient outcomes when this threshold is exceeded and studies showing good tolerance after achieving this target with beta-blockers [7, 11, 45–48]. More recently, several studies conducted in China [28–30, 32] that selected a similar HR target showed good tolerance of the beta-blocker treatment. Therefore, the HR target of < 95 bpm seems appropriate for further comparison and optimal with respect to feasibility and tolerance.

In order to minimize the risk of hemodynamic compromise, patients are only included in the study after at least 24 h of adequate fluid resuscitation and vasopressor support. In addition, landiolol is started at a low dose of 1 mcg/kg/min (as supported by landiolol sepsis [49], postoperative [50], and heart failure studies [51]) and titration is performed conservatively at 20-min intervals (5 half-lives) [35]. The HR target should be reached within the first 24 h, as supported by published data [28–30, 32].

Esmolol is the beta-blocker that has been most frequently evaluated in patients with sepsis, as its pharmacokinetic profile allows for rapid titration when used intravenously [10, 27–29, 31]. However, landiolol has demonstrated a more favorable pharmacokinetic and pharmacodynamic profile than esmolol [52–55]. Landiolol has a faster onset (1 min vs 2 min) and shorter half-life (4 min vs 9 min) than esmolol, which should allow for more rapid titration and enhanced safety [35]. Furthermore, the beta1 selectivity of landiolol is eight times higher than that of esmolol, resulting in a beta1/beta2 ratio of 255 [35]. This provides landiolol with more profound negative chronotropic effects and a lesser degree of negative inotropic and hypotensive action [36–38, 56].

This study will investigate whether HR control using the short-acting beta-blocker landiolol is feasible, safe, and effective in patients with septic shock and persistent tachycardia, and provide a better understanding of the potential role of beta-blockers in this patient population.

## Trial status

Protocol version 3.0 dated 3 January 2018. The first participant was enrolled on 24 February 2018.

## Additional file

Additional file 1: Checklist_SPIRIT_guidelines.pdf, Checklist of the Standard Protocol Items: Recommendations for Interventional Trials (SPIRIT) guidelines. (PDF 129 kb)

## Abbreviations

APACHE II: Acute Physiology and Chronic Health Evaluation II
AV: Atrioventricular
Bpm: Beats per minute
CI: Cardiac Index
CO: Cardiac output
ELWI: Extravascular lung water index
GEDI: Global end-diastolic volume index
HR: Heart rate
ICU: Intensive care unit
LVEF: Left ventricular ejection fraction
MPAP: Mean pulmonary arterial pressure
PAOP: Pulmonary arterial occlusion pressure
NYHA: New York Heart Association
PICCO: Pulse contour cardiac output
SOFA: Sequential Organ Failure Assessment
SPIRIT: Standard Protocol Items: Recommendations for Interventional Trials
TAPSE: Tricuspid annular plane systolic excursion
VTI: Velocity time integral
